# Knowledge and Factors Associated With the Practice of General Health Checks Among Adults in the UAE: A Cross-Sectional Study

**DOI:** 10.7759/cureus.103278

**Published:** 2026-02-09

**Authors:** Arwa Ahmed, Raniya Al-Janabi, Marwa Hussain, Abdulla Alsharif, Abdelrahman Elashry, Sarra Shorbagi

**Affiliations:** 1 College of Medicine, University of Sharjah, Sharjah, ARE; 2 Family Medicine, University of Sharjah, Sharjah, ARE

**Keywords:** cross-sectional study, general health checks, healthcare utilization, non-communicable diseases (ncds), preventive healthcare, united arab emirates

## Abstract

Background: General health checks (GHCs) are preventive encounters with asymptomatic individuals aimed at early disease detection and risk assessment, yet evidence supporting their overall effectiveness remains mixed. In the United Arab Emirates (UAE), where non-communicable diseases account for a substantial proportion of morbidity and mortality, preventive health services are increasingly emphasized.

Aim: This study aimed to assess knowledge and practices related to general health checks among adults in the UAE and to identify factors associated with their utilization.

Methods: A cross-sectional study was conducted between January and March 2023 using a self-administered online questionnaire distributed via snowball sampling. Adults aged 18 years and above residing in the UAE were included, and participants who did not speak Arabic or English were excluded. The questionnaire assessed sociodemographic characteristics, health-related factors, knowledge of GHCs, and self-reported GHC practice, guided by Andersen’s Behavioral Model of Health Services Use. Descriptive statistics, chi-square tests, and binary logistic regression were performed. Analyses were conducted using IBM Corp. Released 2023. IBM SPSS Statistics for Windows, Version 29. Armonk, NY: IBM Corp. at a 5% significance level.

Results: Among 422 participants (308 females, 114 males; 24.9% UAE nationals, 75.1% non-nationals), 62.6% practiced GHCs. The median knowledge score on GHCs was 33.33 with an interquartile range (IQR) of 27.7-44.4. Logistic regression indicated participants aged 35-44 and ≥45 were more likely to practice GHCs than those aged 18-24 (odds ratio [OR] = 4.545, 95% confidence interval [CI] 1.892-10.921, p<0.001; OR = 3.077, 95% CI 1.215-7.797, p = 0.018). Participants with healthcare insurance or a family history of chronic conditions were also more likely to practice GHCs (OR=2.059, 95% CI 1.164-3.642, p=0.013; OR=2.382, 95% CI 1.432-3.962, p<0.001).

Conclusions and recommendations: These findings suggest that participation in GHCs in the UAE is influenced more by demographic and health system factors than by knowledge, highlighting a gap between awareness and informed preventive behavior. Further research is needed to examine the quality, motivations, and long-term outcomes associated with GHCs participation.

## Introduction

General health checks (GHCs) are defined as encounters between health professionals and asymptomatic individuals that are not prompted by specific health complaints, during which multiple assessments or screening tests are conducted to evaluate overall health status [1]. The primary aims of GHCs are the early identification of disease or risk factors, prevention of future illness, and reassurance regarding an individual’s health [1]. In contrast to targeted population screening programs that focus on specific diseases within defined risk groups and illness-related clinical visits prompted by symptoms, GHCs involve broader, non-symptom-driven assessments that may include multiple preventive evaluations delivered opportunistically or periodically. According to UAE guidelines, GHCs are recommended starting as early as 18 years of age [2]; typical components may include measurement of blood pressure, body mass index (BMI), blood glucose, lipid profiles, and selected age- or sex-specific screening tests, depending on local practice and guidelines [3].

Despite their widespread use, evidence regarding the effectiveness of GHCs remains mixed. Large randomized trials and meta-analyses, including a Cochrane systematic review, have shown that general health checks do not result in significant reductions in all-cause mortality or overall morbidity [1]. However, this lack of overall mortality benefit does not negate the value of specific evidence-based components that may be delivered within the context of GHCs. Targeted screening interventions, such as mammography, have demonstrated effectiveness in reducing the incidence of late-stage breast cancer through early detection and timely treatment; these benefits are attributable to the screening intervention itself rather than to general health checks as a comprehensive strategy [4].

GHCs have also been criticized for potential disadvantages when applied indiscriminately to low-risk populations [1,3]. Evidence suggests that GHCs may lead to overdiagnosis, false-positive findings, unnecessary follow-up investigations, psychological distress, and inefficient use of healthcare resources [1]. These concerns have led to recommendations favoring targeted, risk-based preventive screening over routine comprehensive health checks for asymptomatic individuals [3].

The relevance of general health checks (GHCs) is particularly evident in the United Arab Emirates (UAE), where non-communicable diseases (NCDs) accounted for approximately 77% of all deaths in 2018, according to the World Health Organization’s country profiles on NCDs [5]. In the same report, an estimated 17% of deaths in the UAE occurred prematurely (before age 70), primarily due to cardiovascular diseases, cancer, diabetes, and chronic respiratory conditions [5]. In response to this high NCD burden, preventive services and health screening have become central components of public health strategies within the UAE healthcare system [6,7].

Several national and emirate-level initiatives illustrate how GHC-related services are operationalized in practice. The Abu Dhabi Public Health Center’s comprehensive screening program (IFHAS) offers tailored, periodic health evaluations that include cardiovascular risk assessment and selected cancer screenings, aiming to support early detection and chronic disease management [6]. Similarly, national initiatives launched by the Ministry of Health and Prevention (MOHAP) promote periodic health screening and cancer screening within primary healthcare settings, targeting conditions such as diabetes, hypertension, and selected malignancies [7]. The frequency and specific components of these evaluations vary by program, age group, and risk profile. Together, these initiatives reflect the country’s emphasis on preventive care rather than a single nationally standardized GHC framework.

Beyond clinical outcomes, some studies suggest that preventive health encounters may enhance patient engagement and patient-provider communication; however, evidence that general health checks (GHCs) consistently reduce healthcare utilization or improve long-term adherence remains inconsistent [8]. Conceptually, health knowledge is often viewed as a prerequisite for engaging in preventive practices, as it underpins risk perception, perceived benefits, and informed decision-making [9]. Nevertheless, empirical evidence indicates that knowledge alone does not reliably translate into preventive action, reflecting a complex and context-dependent relationship between knowledge and behavior [10].

Participation in GHCs remains variable and is influenced by multiple barriers. Patient-related factors include limited knowledge, personal beliefs, and demographic characteristics [8], while the presence of chronic disease has been identified as one of the strongest predictors of participation [10]. Healthcare system factors, such as staff shortages, overcrowded primary care facilities, and limited resources, further contribute to reduced uptake [11]. Additional determinants, including health insurance coverage, existing chronic conditions, and engagement in physical activity, have been positively associated with attendance at health checks [12].

In the UAE, existing research has largely focused on knowledge and practices related to specific cancer screening programs, including breast and cervical cancer screening [13] and colorectal cancer screening [14]. However, broader aspects of GHCs among asymptomatic adults remain underexplored. Evidence from neighboring countries has suggested low participation in general health evaluations despite relatively high levels of health knowledge, indicating a potential gap between awareness and practice [15]. Given regional similarities in healthcare delivery and preventive health priorities, examining these patterns within the UAE context is of particular interest. Therefore, this study aims to assess knowledge and practices related to general health checks among adults in the UAE and to identify factors associated with their uptake using Andersen’s Behavioral Model of Health Services Use.

## Materials and methods

Study design, setting, and population

This cross-sectional study was conducted between January and March 2023 in the United Arab Emirates (UAE). The target population included adults aged 18 and above living in the UAE. An estimate of the sample size was calculated using Cochran's formula, with p set at 0.5 to maximize sample size, an error margin of 5%, and Z = 1.96 for a 95% confidence interval, resulting in a required sample of 385 participants. A total of 428 responses were received, of which six were removed due to duplicates. All questionnaire items were set as mandatory on Google Forms, which prevented submission of incomplete responses.

Ethical consideration

The University of Sharjah Research Ethics Committee (REC-23-02-18-10-S) approved this study. Participants received an information sheet and were informed that completing the questionnaire indicated consent. Although the research was anonymous, confidentiality was maintained by securely storing data on an encrypted drive accessible only to the researchers and supervisor. The inclusion criteria are adults living in the UAE above the age of 18. Participants who did not speak Arabic or English were excluded.

Data collection

Data were collected using a self-administered 32-item multiple-choice online questionnaire, administered in English and Arabic language. The questionnaire was developed based on Andersen’s Behavioral Model of Health Services Use [16], a widely applied conceptual framework, and is available for unrestricted academic use without licensing requirements. This model is used to examine factors influencing healthcare utilization, including general health checks, by categorizing them into three main components: predisposing, enabling, and need factors.

**Figure 1:**
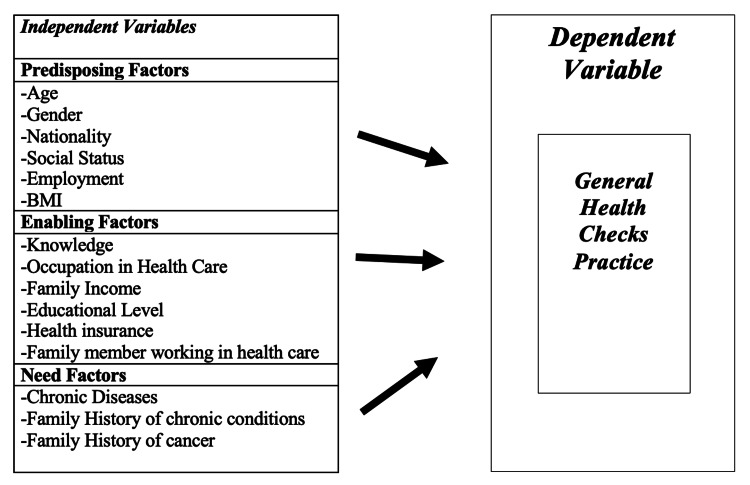
The Andersen model to predict the practice of general health checks (GHC) Conceptual framework of factors influencing general health check (GHC) practice, based on the modified Andersen Behavioral Model. Adapted from Lee, H. Y. [17]

The questionnaire comprised two main sections assessing independent and dependent variables. Independent variables were categorized according to Andersen’s model (Figure 1). Predisposing factors included sociodemographic characteristics such as age, gender, nationality, and income. Enabling factors included education level, knowledge, health insurance coverage, and having a family member working in healthcare. Need factors reflected perceived health status, presence of chronic conditions, and family history of chronic conditions [16].

Chronic conditions were reported by participants and included diabetes mellitus, hypertension, heart conditions (including myocardial infarction, angina, or congestive heart failure), long-term respiratory diseases (such as asthma, emphysema, or chronic bronchitis), arthritis, and rheumatologic conditions. Family history of chronic disease was assessed using the same categories.

The dependent variable practice of GHCs was assessed through participant self-report. Participants were asked whether they had undergone a general health check, defined in the questionnaire as contacts between health professionals and asymptomatic individuals that are not prompted by specific complaints, during which multiple assessments or screening tests are performed to evaluate overall health status.

Knowledge was assessed using an 11-item questionnaire adapted from a previously published open-access study conducted in Saudi Arabia [10]. Three items were modified to align with the UAE context and updated according to national guidelines [2]. The adapted items were reviewed by two independent physicians (one in family medicine and one in internal medicine) to ensure content validity and clinical accuracy. The questionnaire was piloted in a small group to assess clarity and completion time, after which additional definitions were incorporated to improve participant understanding. Internal consistency of the knowledge scale was assessed using Cronbach’s alpha [17].

The questionnaire was distributed via social media using a snowball sampling method. This approach has been widely used in preventive health research to access diverse community samples, though it may limit representativeness. It was selected for logistical feasibility, allowing for efficient and cost-effective dissemination and a larger sample size. For participant recruitment, initial recruitment seeds consisted of healthcare workers, university students, and community members, who were invited to complete the online survey and disseminate it within their professional and social networks. Primary dissemination channels included WhatsApp, email, and other social media platforms, consistent with common approaches used in online snowball sampling studies.

Statistical analysis and coding

Data analysis was performed using IBM Corp. Released 2023. IBM SPSS Statistics for Windows, Version 29. Armonk, NY: IBM Corp. All demographic and categorical variables were coded using binary (0/1) coding. Knowledge scores were calculated as the percentage of correctly answered items, with correct answers scored as 1 and incorrect answers as 0. As this is a newly adapted questionnaire, the knowledge score variable is treated as continuous for descriptive and analytic purposes. Descriptive statistics were used to summarize categorical and continuous variables. Bivariate associations between independent variables and routine GHC practice were assessed using the chi-square test. Binary logistic regression was employed to examine the association between the knowledge score and GHC practice while adjusting for confounding factors identified in bivariate analyses. Assumptions of logit linearity and absence of multicollinearity were checked prior to analysis. Standardized residuals were inspected for outliers. P-values less than 0.05 were considered statistically significant.

## Results

**Figure 2:**
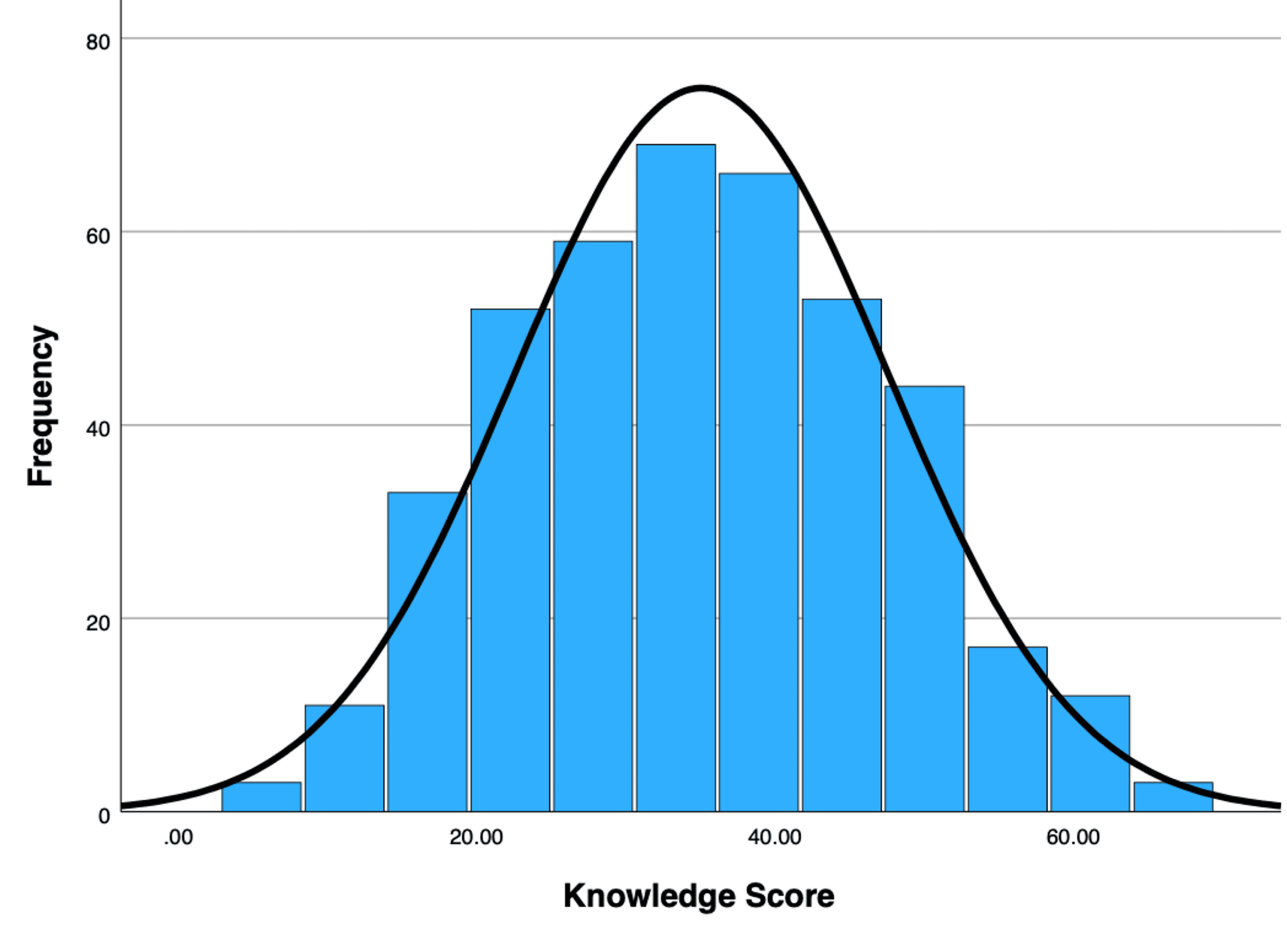
Knowledge scores among the study participants (n = 422)

Of the 428 submitted questionnaires, six duplicate responses were removed. The remaining 422 responses were complete, resulting in a 100% item completion rate and no missing data in the final dataset. Out of the total sample, 264 individuals (62.6%) practice GHCs, and 37.4% don’t practice GHCs. Figure 2 illustrates their knowledge scores regarding routine medical checkups. The mean knowledge score was 35.03 (SD = 12.49), with a median of 33 (IQR 27.7-44.4). The Cronbach’s alpha test value is 0.75, indicating acceptable reliability.

Table 1 presents the demographic characteristics of the sample. Most respondents were aged 18-24 years (62.1%). The majority were female (73%) and UAE non-locals (75.1%). Over half reported holding a high school diploma (53.8%), being unemployed (62.1%), and having a monthly income between 10,000 and 19,999 AED (24.2%).

**Table 1:** Demographics of the study participants (n=422)

Variable	N [%]
Age group	
18 - 24	262 [62.1]
25 – 34	59 [14.0]
35 – 44	57 [13.5]
45+	44 [10.4]
Gender	
Female	308 [73.0]
Male	114 [27.0]
Nationality	
UAE local	105 [24.9]
UAE non-local	317 [75.1]
Level of Education	
High school diploma or less	230 [54.5]
Bachelor’s degree	156 [37.0]
Postgraduate	36 [8.5]
Employment Status	
Full-time employed	133 [31.5]
Part-time employed	27 [6.4]
Unemployed	262 [62.1]
Monthly Family income	
Less than 4,999 AED	50 [11.8]
5,000-9,999 AED	55 [13.0]
10,000-19,999 AED	102 [24.2]
20,000 -29,999 AED	72 [17.1]
30,000 -39,999 AED	42 [10.0]
Above 40,000 AED	101 [23.9]
health care insurance	
Yes	341 [80.8]
No	81 [19.2]

Table 2 summarizes participants’ self-reported medical and family histories. Diabetes mellitus was the most common reported chronic condition (7.8%), followed by hypertension (6.6%). Cancer was the least frequently reported condition, affecting only two participants (0.5%). Additionally, nearly two-thirds of participants (64%) indicated a family history of chronic conditions, while 39.8% reported a family history of cancer.

**Table 2:** Medical and family history of the study participants (n=422)

Condition	N [%]
Medical history of chronic disease	
Diabetes mellitus	33 [7.8]
Hypertension	28 [6.6]
Heart conditions (such as heart attack, angina, or congestive heart failure)	38 [9.0]
Long-term lung diseases (ex: asthma, emphysema, chronic bronchitis)	3 [0.7]
Arthritis or rheumatism	9 [2.1]
Cancer	2 [0.5]
Family history	
Family history of cancer	168 [39.8]
Family history of chronic conditions	270 [64.0]

Table 3 outlines the types of examinations and tests reported by participants as part of their GHCs. The most frequently endorsed items were blood pressure measurement (57.6%), height and weight measurement (55.9%), and fasting blood sugar testing (45.5%). In contrast, the least commonly reported components were breast cancer screening (4.5%) and cervical cancer screening (2.1%).

**Table 3:** Types of assessments and tests included in GHCs reported by study participants (n = 422)

Contents of GHCs done	N [%]
Blood pressure measurement	243 [57.6]
Height and weight measurement	236 [55.9]
Fasting blood sugar	192 [45.5]
Blood lipids profile	156 [37.0]
Bone mass density	21 [5.0]
Breast cancer screening	19 [4.5]
Cervical cancer screening	9 [2.1]

A chi-square test of independence (Table 4) revealed significant associations between the practice of GHCs and several participant characteristics. Engagement in GHCs varied significantly by age group (χ²(3) = 16.59, p < .001), nationality (χ²(1) = 5.757, p = .016), having health insurance (χ²(1) = 20.373, p < .001), and family history of chronic conditions (χ²(1) = 12.822, p < .001). Insured participants, UAE non-locals, and those with a family history of chronic conditions were more likely to report practicing GHCs. No significant associations were observed with gender.

**Table 4:** Bivariate analysis of GHC practice by sociodemographic and family history characteristics (n = 422)

Routine Medical Checkup Practice	Total	Yes	No	Chi-square	p-value
		N (%)	N (%)		
Age group					
18 – 24	262	152 (58.0)	110 (42.0)	16.596	< .001>
25 – 34	59	32 (54.2)	27 (45.8)		
35 – 44	57	47 (82.5)	10 (17.5)		
45+	44	33 (75.0)	11 (25.0)		
Gender					
Female	308	188 (61.0)	120 (39.0)	1.125	.289
Male	114	76 (66.7)	38 (33.3)		
Nationality					
UAE local	105	76 (72.4)	29 (27.6)	5.757	.016
UAE non-local	317	188 (59.3)	129 (40.7)		
Healthcare insurance					
Yes	341	231 (67.7)	110 (32.3)	20.373	
No	81	33 (40.7)	48 (59.3)		
Family history of chronic conditions					
Yes	270	186 (70.5)	84 (53.2)	12.822	
No	152	78 (29.5)	74 (46.8)		

Binary logistic regression was performed to identify independent predictors of GHC practice (Table 5). Model assumptions for logit linearity and absence of multicollinearity were met. Five outliers were retained, as their exclusion did not materially alter model estimates. The overall model was statistically significant and explained approximately 15-21% of the variance in GHC practice, correctly classifying 70.6% of cases.

**Table 5:** Logistic regression model of predictors of GHC practice (n = 422) GHC: General health check

Co-variates	N	OR	95% CI	p-value
Age				
18-24	262	(Ref)	(Ref)	(Ref)
25-34	59	0.948	(0.449- 2.005)	0.890
35-44	57	4.545	(1.892-10.921)	<0.001
45+	44	3.077	(1.215-7.797)	0.018
Nationality				
UAE local	105	1.668	(0.958-2.910)	0.071
UAE non-local	317	(Ref)	(Ref)	(Ref)
Gender				
Female	308	(Ref)	(Ref)	(Ref)
Male	114	1.454	(0.853-2.478)	
Health Insurance				
Yes	341	2.059	(1.164-3.642)	0.013
No	81	(Ref)	(Ref)	(Ref)
Family History of chronic conditions				
Yes	270	2.382	(1.432-3.962)	<0.001
No	152	(Ref)	(Ref)	(Ref)
Knowledge Score	422	1.008	(0.989-1.028)	0.413

After adjustment, older age (35-44: OR = 4.545, 95% CI 1.892-10.921, p < .001; 45+: OR = 3.077, 95% CI 1.215-7.797, p = .018), having health insurance (OR = 2.059, 95% CI 1.164-3.642, p = .013), and reporting a family history of chronic conditions (OR = 2.382, 95% CI 1.432-3.962, p < .001) remained significant predictors of GHC engagement. Knowledge scores and nationality were not significant predictors in the adjusted model.

## Discussion

This study investigated knowledge and practice of general health checks (GHCs) among adults in the UAE. Overall, 62.6% of participants reported engaging in at least one GHC, while knowledge scores regarding GHCs showed a median of 33 (IQR 27.7-44.4), not significantly associated with GHC practice in the adjusted regression model (p = 0.413), indicating that higher awareness did not translate into greater engagement. These findings suggest that participation in preventive health checks may be influenced more by demographic, systemic, or personal health factors than by knowledge alone. Comparable evidence from Saudi Arabia reported low participation in routine medical checkups despite higher levels of knowledge [15], supporting the notion that awareness does not necessarily drive preventive behavior. 

When compared to regional data, the prevalence of GHC engagement in this study appears relatively high. For instance, in Saudi Arabia, only 34.3% of adults aged ≥30 reported participating in routine medical checkups [15]. Another Saudi study showed low GHCs engagement: 75% of Saudi medical students have never had a routine check-up [12]. Similarly, studies in other Middle Eastern contexts have documented suboptimal uptake despite widespread availability of primary care services, often citing barriers such as limited awareness, time constraints, or cultural norms [10,12]. Internationally, participation rates vary considerably: approximately 50-53% of adults in Germany and Japan report routine health checkups [18,19]. The practice rate in our study may reflect broad insurance coverage and growing health awareness in the UAE, supported by recent data from the Dubai Health Authority stating that over 4.47 million individuals are covered under Dubai’s health insurance system in 2023 [20].While these comparisons provide context, it is important to note significant methodological and structural differences across studies, including age distribution, operational definitions of GHCs, frequency of checkups, insurance coverage, and national screening policies. Consequently, direct comparisons should be interpreted cautiously, with emphasis on descriptive trends rather than definitive conclusions about relative performance.

Despite the higher prevalence of GHC practice observed in this study, knowledge about their purpose and components remained limited. This pattern mirrors findings from Saudi Arabia [15] and suggests a knowledge-practice disconnect, where individuals may attend health checks opportunistically or due to external prompts, such as employment requirements, insurance-mandated assessments, or physician advice, rather than informed, proactive health decisions. Although our study did not directly measure the motivations for attending GHCs, the observed discrepancy between low knowledge and relatively high participation highlights the need to distinguish behavioral engagement from health literacy when evaluating preventive care utilization. 

Consistent with Andersen’s behavioral model [16], several factors were significantly associated with GHC engagement. Older age, having health insurance, and reporting a family history of chronic conditions were independently linked to a higher likelihood of attending GHCs. Age likely reflects predisposing factors, as older adults may perceive greater vulnerability to illness and are more likely to be targeted by age-specific screening recommendations. Health insurance represents an enabling factor, facilitating access to preventive services and reducing financial barriers, consistent with international evidence that insured individuals utilize preventive care at higher rates than uninsured populations [21]. Family history of chronic conditions reflects perceived or actual need, as awareness of personal risk may motivate individuals to participate in GHCs even in the absence of symptoms. These findings support prior research in Gulf Cooperation Council populations, where age, insurance, and need factors were consistently stronger predictors of preventive care utilization than demographic or educational characteristics [22,23].

Within the framework of Andersen’s Behavioral Model, not all predisposing and enabling factors examined were significantly associated with General Health Checkup (GHC) utilization. Variables such as education level, income, and employment status did not demonstrate independent associations with GHC practice in the multivariable analysis. This finding suggests that, in this sample, structural and system-level factors may play a more prominent role in GHC utilization than individual socioeconomic characteristics. The absence of significant associations for these variables does not imply that they are unimportant; rather, it may reflect contextual features of the UAE healthcare system, including widespread insurance coverage in certain emirates, employer-sponsored health benefits, and relatively accessible private healthcare services, which may attenuate traditional socioeconomic gradients in preventive care utilization. Additionally, the non-significance of these variables may be influenced by the study’s predominantly young, insured, and employed sample, as well as limited variability within certain socioeconomic categories.

Limitations

Several limitations should be acknowledged. First, the cross-sectional study design limits causal inference, and the observed associations may be influenced by unmeasured confounding factors or reverse causation. Second, the knowledge assessment relied on a newly adapted questionnaire and was used for descriptive purposes only, as no established cut-off values exist to define adequate or inadequate knowledge. Third, data were self-reported and collected through an online snowball sampling method, which may introduce selection bias, social desirability bias, and recall bias. Additionally, the study sample was predominantly young, female, and non-national, potentially limiting the generalizability of the findings to the broader adult population in the United Arab Emirates. Fourth, the study did not assess participants’ motivations for attending GHCs or the specific contexts in which they occurred (e.g., employer-mandated assessments or opportunistic screenings), which restricts interpretation of the underlying behavioral drivers of participation. Fifth, although the overall sample size was adequate to detect moderate associations for common variables, the study may have been underpowered to identify small effect sizes or associations involving low-prevalence conditions, such as specific chronic diseases. Consequently, nonsignificant findings should be interpreted with caution and should not be considered evidence of no association. In addition, the pseudo-R² values indicated a weak overall model fit, suggesting that the included predictors explained only a limited proportion of variability in GHC utilization; therefore, the identified associations should be interpreted cautiously and not overestimated in terms of predictive value.

Finally, comparisons with regional and international studies should be interpreted cautiously due to differences in operational definitions of GHCs, healthcare access, screening policies, and population characteristics.

Implications

These results carry several implications for public health and preventive care strategies in the UAE. First, interventions focused solely on increasing knowledge about GHCs may have limited impact on uptake, particularly among younger adults who reported lower engagement. Second, systemic factors such as health insurance coverage and accessibility appear to play a central role in facilitating participation, suggesting that policies ensuring universal coverage and easy access to preventive services may sustain higher utilization rates. Third, understanding the motivations behind GHC attendance, whether opportunistic, employment-mandated, or proactive, could inform the design of targeted programs that encourage both informed engagement and comprehensive preventive assessments. While this study cannot determine causal relationships, these findings provide a foundation for hypothesis-driven research exploring drivers of preventive care behavior and the effectiveness of GHCs in improving health outcomes.

## Conclusions

This study shows that engagement in general health checks among UAE adults is relatively common, but knowledge regarding their purpose and components remains limited. Participation was associated with older age, insurance coverage, and family history of chronic conditions, whereas knowledge scores were not predictive. These findings emphasize the descriptive nature of knowledge scores and caution against overinterpreting engagement as evidence of informed preventive behavior. Future research should explore the motivations behind GHC attendance, assess the quality and completeness of screenings received, and evaluate whether regular participation translates into improved long-term health outcomes.

## Appendices

Knowledge, practices, and predictive factors related to general health checks among adults in the UAE

We are a group of medical students at the University of Sharjah conducting a research project about the “Knowledge and Practices and Predictive Factors Toward General Health Checks among Adults in the UAE" The purpose of this study is to assess the knowledge, practices, and predictive factors related to routine medical checkups among adults in the UAE. Your participation is strictly voluntary. If you agree to participate, you will be asked to fill out a questionnaire that should take 6 to 8 minutes of your time. There are no risks associated with participation in this study. We assure you that your responses will be confidential and will be used only for research purposes. Hence, after submitting your response, it cannot be removed from the database. There will be no benefits gained from participation in this study. If you have any questions regarding this study, please feel free to contact Arwa Mamoon at u20100974@sharjah.ac.ae or Raniya Shamel at u20102751@sharjah.ac.ae or our research supervisor -Dr. Sarra Shorbagi, at ssaeed@sharjah.ac.ae or 06/5057229. For any further ethical concerns, you may contact the head of the Research Ethics Committee, University of Sharjah -Dr. Suhail Al Amad at rec@sharjah.ac.ae -06/5057304. Filling this questionnaire indicates your agreement to participate in the study.

* Indicates required question

1. Please enter the last three letters of your family name *

2. Please enter the last three digits of your phone number *

3. Select your age group: *Mark only one oval.

1. 18-24

2. 25-34

3. 35-44

4. 45-54

5. 55 and above

4. Specify your gender: *Mark only one oval.

1. Female

2. Male

5. Enter your height in (cm): *

6. Enter your weight in (kg): *

7. What is your nationality? *Mark only one oval.

1. UAE local

2. UAE non-local

8. What is your social status? *Mark only one oval.

1. Married

2. Single

3. Divorced

4. Widowed/Widower

9. Which level of education did you complete? *Mark only one oval.

1. Did not complete school

2. High school diploma

3. Bachelor’s degree

4. Postgraduate

10. What is your current employment status? *Mark only one oval.

1. Full-time employed

2. Part-time employed

3. Unemployed

11. Are you a health care provider? *Mark only one oval.

1. Yes

2. No

3. Not applicable (not working)

12. Select your family income Per month: *Mark only one oval.

1. Less than 4,999 AED

2. 5,000-9,999 AED

3. 10,000-19,999 AED

4. 20,000 -29,999 AED

5. 30,000 -39,999 AED

6. Above 40,000 AED

13. Do you have a first-degree family member working in healthcare? *Mark only one oval.

1. Yes

2. No

14. Do you have health care insurance? *Mark only one oval.

1. Yes

2. No

15. Does the insurance cover the general health check costs? *Mark only one oval.

1. Yes

2. No

3. Not Sure

4. Not applicable (I don’t have health insurance)

16. Select all health conditions you currently have or had: *Tick all that apply.

1. High blood sugar (diabetes mellitus)

2. High blood pressure (Hypertension)

3. Heart conditions (such as heart attack, angina, or congestive heart failure)

4. Long term lung diseases (ex: asthma, emphysema, chronic bronchitis)

5. Arthritis and or rheumatism

6. Cancer

7. None

17. Do you Have Any family history of cancer? *Mark only one oval.

1. Yes

2. No

18. Do you have any family history of chronic conditions? *Chronic conditions such as high blood sugar (diabetes mellitus), High blood pressure (Hypertension), Heart conditions (such as heart attack, angina, or congestive heart failure), Long-term lung diseases (e.g., asthma, emphysema, chronic bronchitis), Arthritis and or rheumatism. Mark only one oval.

1. Yes

2. No

Knowledge

19. How often should a general health check be done? *Mark only one oval.

1. Every month

2. Every 6 months

3. Every year

4. Depends on the age*

5. I do not know

20. What does a general health check include?* (select all that apply)

1. Blood pressure measurement*

2. Height and weight measurement*

3. Fasting blood sugar test*

4. Blood lipids profile test*

5. Bone mass density test*

6. Breast cancer screening*

7. Cervical cancer screening*

8. Prostate cancer screening

21. Which of the following is a Body Mass Index indicating morbid obesity? *(BMI is a mathematical formula for identifying a person's normal weight, which is the result of dividing the weight by the square of the height in metres) Mark only one oval.

1. Less than 20

2. Equal to 35

3. More than or equal to 40*

4. I do not know

22. What is the recommended age for men to begin screening blood lipid levels on a routine basis, especially among individuals without known risk factors? Mark only one oval.*

1. 20 years

2. 35 years

3. 45 years*

4. I do not know

23. What is the recommended age for women to begin screening for blood lipid levels on a routine basis, especially among individuals without known risk factors? *Mark only one oval.

1. 20 years

2. 35 years*

3. 45 years

4. I do not know

24. Which of the following do you consider as a normal systolic blood pressure? Mark only one oval.*

1. Less than 90

2. Between 120-129*

3. Between 140-159

4. I do not know

25. When fasting, blood glucose equals 130 mg/dl; this is considered: *Mark only one oval.

1. Low

2. Normal

3. High*

4. I do not know

26. What is the recommended age to begin screening for osteoporosis? *Mark only one oval.

1. 50 years

2. 60 years

3. 65 years*

4. I do not know

27. What is the recommended age to begin blood glucose screening? *Mark only one oval.

1. 30 years*

2. 40 years

3. 50 years

4. I do not know

28. What is the recommended age to initiate breast cancer screening among women with no family history of the disease? *Mark only one oval.

1. 50 years

2. 40 years*

3. 30 years

4. I do not know

29. What is the recommended age to initiate colon cancer screening among men with no family history of the disease?* Mark only one oval.

1. 50 years

2. 40 years*

3. 60 years

4. I do not know

Practices

30. Have you ever had a general health check? (General health checks (GHCs) are defined as contacts between health professionals and asymptomatic individuals that are not prompted by specific complaints, during which multiple assessments or screening tests are performed to evaluate overall health status) Mark only one oval.*

1. Yes

2. No

31. Have you had a general health check in the past year? *Mark only one oval.

1. Yes

2. No

32. Which of the following was done in the general health check you have been to: (select all that applies)

Tick all that apply.*

1. Blood pressure measurement

2. Height and weight measurement

3. Fasting blood sugar test

4. Blood lipids profile test

5. Bone mass density test

6. Breast cancer screening

7. Cervical cancer screening

8. Prostate cancer screening

9. I Have not had a routine medical checkup
